# Evaluation of national trends in the utilization of partial nephrectomy in relation to the publication of the American Urologic Association guidelines for the management of clinical T1 renal masses

**DOI:** 10.1186/1471-2490-14-101

**Published:** 2014-12-17

**Authors:** Michael A Liss, Song Wang, Kerrin Palazzi, Ramzi Jabaji, Nishant Patel, Hak J Lee, J Kellogg Parsons, Ithaar H Derweesh

**Affiliations:** Department of Urology, University of California San Diego Health System, La Jolla, CA USA; UCSD Moores Cancer Center, 3855 Health Sciences Drive, La Jolla, CA 92093 MC 0987 USA

**Keywords:** Kidney cancer, Outcomes, Nephrectomy

## Abstract

**Background:**

Partial nephrectomy has been underutilized in the United States. We investigated national trends in partial nephrectomy (PN) utilization before and after publication of the American Urological Association (AUA) Practice Guideline for management of the clinical T1 renal mass.

**Methods:**

We identified adult patients who underwent radical (RN) or PN from November 2007 to October 2011 in the Nationwide Inpatient Sample (NIS). PN prevalence was calculated prior to (11/2007-10/2009) and after Guidelines publication (11/2009-10/2011) and compared the rate of change by linear regression. We also examined the nephrectomy trends in patients with chronic kidney disease (CKD). Statistical analysis included linear regression to determine point-prevalence of PN rates in CKD patients and logistic regression to identify variables associated with PN.

**Results:**

During the study period, 30,944 patients underwent PN and 64,767 RN. The prevalence PN increased from 28.9% in the years prior to guideline release to 35.3% in the years following guideline release with an adjusted odds ratio (OR) of 1.24 (CI 1.01–1.54; p = 0.049). The rate of PN significantly increased throughout the study period (R^2^ 0.15, p = 0.006): however, the rate of change was not increased after the guidelines. (p = 0.46). Overall rate of PN in patients with CKD did not increase over time (R^2^ 0.0007, p = 0.99).

**Conclusion:**

We noted a 6.4% absolute increase in PN after release of the AUA guidelines on clinical T1 renal mass was published; however, the rate of increase was not likely associated with guideline release. The rate of PN performed is increasing; however, further investigation regarding medical decision-making surrounding PN is needed.

## Background

Radical nephrectomy (RN) had been the standard of care for renal malignancy for nearly 4 decades [1]. However, increasing detection of small renal masses (SRM) has lead to a stage migration of renal masses [2, 3]. Smaller tumors suggest the opportunity perform more nephron sparing surgery; however, large national databases have suggested underutilization of PN [4–7].

Increased knowledge of chronic cardiac and metabolic sequelae of RN, along with data suggesting oncologic equivalence for partial (PN) compared to radical nephrectomy for SRM, has prompted reconsideration of surgical approaches to renal masses [8–14]. In 2009, the American Urological Association (AUA) published evidence-based practices guidelines for the management of clinical T1a renal masses as a guide for clinicians to consider PN to be the reference standard for T1a renal masses, and emphasizing the importance of renal preservation in patients with chronic kidney disease (CKD) [15].

To investigate the potential shorter-term impact of publication of the AUA T1 Renal Mass Guidelines, we analyzed the overall and rate of utilization of PN versus radical nephrectomy (RN) in a national cohort before and after the AUA Guideline release, with a focused analysis of utilization trends of PN in patients with non-dialysis dependent CKD.

## Methods

### Study design

As the primary outcome, we investigated the proportion and rate of PN performed after the release of the AUA T1 Renal Mass Guidelines compared to prior using a publicly available cross-sectional inpatient database (National Inpatient Sample, NIS). Patients were divided into two groups consisting of a 2-year span prior to and 2 year after the release of the AUA T1 Renal Mass Guidelines. A secondary outcome is to investigate the rates of RN vs. PN in patients with non-dialysis dependent chronic kidney disease (CKD) [ICD 9 code for CKD stages 3–4; i.e. glomerular filtration rate (GFR) of 59–31 and 30–15 ml/min/1.73 m^2^, respectively)] in both time periods. Additionally, we investigated patient and external factors associated with performance of RN vs. PN in order to identify potential changes in disparities regarding utilization of nephron sparing surgery and the possible factors associated with patient selection.

### Database cohort: the nationwide inpatient sample

We analyzed data from November 2009 to October 2011 in the Nationwide Inpatient Sample (NIS), which is part of the Healthcare Cost and Utilization Project (HCUP) sponsored by the Agency for Healthcare Research and Quality (AHRQ). The database is de-identified and publically available; therefore, is exempt from formal IRB review. It is the largest all-payer inpatient care database that is publicly available in the United States, containing data from 5 to 8 million hospital stays per year. In 2010, data included 1,056 hospitals located in 42 states, making up a 20% stratified sample of U.S. hospitals [12]. Inpatient stay records include clinical and resource use information available from discharge abstracts. Weighted sampling allows estimates for national trends. The data is publically available and de-identified so no patient consent was needed for this study. The NIS is available for purchase at http://www.hcup-us.ahrq.gov/nisoverview.jsp.

### Variables

We focused on age, Charlson comorbidity index, and CKD as specific variables, which are emphasized in with the AUA clinical T1 Renal Mass Guidelines. However, CKD coding in the NIS can be non-specific for stage depending on how the medical history was coded. Therefore, we will use the CKD codes for a general term CKD with subsequent sub analysis of specifically CKD stage 3 and 4 (eGFR range of 15–59). Other abstracted exposure variables included race, gender, treatment year, income by zip code, geographic region of treatment (Northeast, South, Midwest, and West), hospital renal surgery volume, insurance type, the hospital size, hospital location (rural vs. urban), and hospital type (teaching versus non-teaching).

### Case selection

#### General

We identified all hospital admissions in patients older than 18 years with a primary or secondary procedure code for PN (PN) (55.4) or radical nephrectomy (RN) (55.5, 55.51, 55.52 and 55.54) from November 2007 to October 2011.

#### Oncologic extent

The NIS does not contain tumor size or disease specific staging. Additionally, patients with locally advanced, locoregional or metastatic disease are more likely to undergo RN. Therefore, we excluded patients undergoing concomitant surgeries suggestive of advanced disease, including splenectomy (41.4, 41.42, 41.43), liver resection (50.2, 50.21-26, 50.29, 50.3), pancreas resection (52.5, 52.51-53, 52.59, 52.6, 52.7), bowel or colon resection (45.5, 45.50-52, 45.61-62, 45.7, 45.71, 45.73-76, 45.79, 45.8, 45.81-83), or thrombectomy with vascular reconstruction (37.10, 38.05, 38.07, 38.45, 38.47, 38.65, 38.67, 38.75, 38.77, 38.87, 39.6, 39.61, 39.63, 39.66) to reduce the potential for bias by burden of disease.

#### Other kidney surgery

The ICD-9 procedure code for nephrectomy does not distinguish between radical and simple operations. Consequently, we excluded all patients with a concomitant ICD-9 diagnosis code for kidney donor (V59.4), and infectious etiologies including acute and chronic pyelonephritis (590.0, 590.00, 590.01, 590.1, 590.10 and 590.11) and renal/perinephric abscess (590.2) since these diagnoses might represent the indication for performing the procedure. We also excluded those patients with a diagnosis of renal pelvic or ureteral tumor (189.1 or 189.2) since nephrectomy and nephroureterectomy utilize the same ICD-9 procedure code. We also excluded autosomal dominant polycystic kidney disease (753.12, 753.13, 753.14) since the diagnosis might affect pre-operative renal function as well as surgical indication and selection.

#### Comorbidity and chronic kidney disease

Co-morbid conditions assessed include hypertension (Dx CCS 98, 99), diabetes (Dx CCS 49, 50) and obesity (ICD-9 278.0, 278.01, 278.00). A subgroup analysis of patients with a discharge ICD-9 diagnosis of chronic renal insufficiency (585x) was performed. We divided patients with a specified CKD stage into those with end stage renal failure (ESRF) requiring dialysis (CKD stage 5, ICD-9 585.5). CKD 6 is an unspecified diagnosis code of 585.6; however, most patients fell into this category possibly due to coding indiscretion to CKD stage.

### Statistical analysis

The 20% NIS sample was weighted to estimate all national inpatient stays and used for all calculations. The primary outcome of proportion of PN performed 2 years before (November 2007-October 2011) and 2 years following (November 2009-October 2011) publication of Guideline for management of the clinical T1 renal mass (AUA Renal Mass Guideline) was examined by the Chi Squared test. The secondary outcome was change in rates of PN over the two time periods were calculated using the total number of inpatients in each year creating simple linear regression models to approximate the change in procedure incidence over time (months). The proportion of PN performed was calculated by the number of partials performed divided by the total number of nephrectomies (RN plus PN). Each time period will have a regression statistical value then the slopes are compared using interactions between the regression lines.

Demographics, clinical and hospital characteristics were compared between groups using chi-squared (Rao & Scott second order correction), ANOVA, and Student t tests (using Bonferroni correction for inter-group comparisons). We grouped patients based on RN vs. PN as well as pre vs. post AUA T1 Renal Mass Guidelines recommendation to examine demographic differences. Because renal mass treatment may be influenced by age we included age as a continues variable as well as a binary variable at age 60 as this was the median age of partial nephrectomy in the AUA guidelines [15]. Additionally, racial differences are compared due to recent evidence that race and gender may influence surgical decision making [16, 17].

Subsequently, we performed multivariable analysis using binary logistic regression models to examine the influence of age, race, sex, co-morbid disease and hospital characteristics on surgery selection; only variables that reached statistical significance in the multivariate model were ultimately included in the final models. SVY coding in STATA v 11.1 (StataCorp, College Station, TX) was used to account for NIS sampling methodology, and probability of type I error was defined a priori as α = 0.05.

## Results

### Population characteristics for partial nephrectomy pre and post guidelines

We identified 95,711, patients undergoing either PN or Radical Nephrectomy (RN) in the United States from November 2009 to October 2011. We compared 30,944 patients who received a PN to 64,767 who received a RN (Table 1). A significantly higher proportion of PN was performed after 2 years after AUA Renal Mass Guideline publication compared to two years prior guidelines were published than prior (35.3 % vs. 29%; p < 0.0001). Patients with CKD who underwent PN was not significantly increased post-guidelines compared to pre-guideline (7.3% vs. 7.9%; p = 0.2997). More specifically, 26.3% (626/2376) PN were performed prior to guideline publication with Stage III/Stage IV CKD compared 28.2% (970/3439) to post-Guideline publication which corresponds to a non-significant increase (p = 0.126).

**Table 1:** **Patient demographics of partial nephrectomy before and after the publication of the T1a renal AUA guidelines recommendations**

Demographic	Before Guidelines	After Guidelines	***p-value***
	N = 27357 (PN)	N = 34336 (PN)	
	N = 94457 (PN + RN)	N = 97314 (PN + RN)	
Mean Age ± SE	58.2 ± 0.2	58.4 ± 0.2	0.3888
Age Group			0.3325
<60	14024(51.3) /44364	17228(50.2) /45978	
≥60	13333(48.7) /50093	17108(49.8) /51336	
Gender			0.1857
Male	15558(57.1) /53780	20015(58.4) /56715	
Female	11672(42.9) /40344	14245(41.6) /40406	
Race			<0.0001
Caucasian	16714(61.1) /57803	23530(68.5) /66065	
African-American	2059(7.5) /7244	3190(9.3) /9575	
Hispanic	1540(5.6) /6125	2489(7.3) /7609	
Asian	440(1.6) /1681	567(1.7) /1752	
Other	6604(24.1) /21604	4558(13.3) 12313	
Income (Zip Code)			0.8705
<$50,000	12000(45.1) /44943	15053(44.7) /46743	
≥$50,000	14609(54.9) /47486	18634(55.3) /48732	
Hypertension	15427(43.6) /56001	20009(58.3) /59732	0.0842
Diabetes	5702(20.8) /20288	7998(23.3) /23219	0.0056
Obesity	3493(12.8) /10846	5180(15.1) /13513	0.0114
Chronic Kidney Disease	1986(7.3) /12058	2697(7.9) /13685	0.2997
CKD Stages			0.6560
Stage1	10(0.5) /25	30(1.1) /75	0.2353
Stage2	83(4.2) /353	124(4.6) /439	0.5822
Stage3	480(24.2) /1770	766(28.4) /2620	0.1144
Stage4	146(7.3) /606	204(7.6) /819	0.6660
Stage5/ESRD	15(0.8) /182	24(0.9) /163	0.7484
Stage unspecified	1252(63.0) /9121	1548(57.4) /9568	0.9392
Charlson Comorbidity Index			0.3327
0	16780(61.3) /52204	20528(59.8) /53063	
1	6763(24.7) /21901	8965(26.1) /23394	
2	1948(7.1) /7365	2375(6.9) /7594	
3+	1866(6.8) /12988	2468(7.2) /13262	
Insurance			0.8561
Private	15224(55.8) /45206	18797(54.9) /45917	
Medicare	9018(33.0) /37728	11416(33.3) /38316	
Medicaid	1561(5.7) /5794	2014(5.9) /6464	
Other	1500(5.5) /5565	2031(5.9) /6352	
Hospital Bed Number			0.3397
Small	2350(8.8) /8298	3077(9.2) /8808	
Medium	5742(21.5) /20598	5766(17.2) /18948	
Large	18569(69.6) /63430	24696(73.6) /67702	
Hospital Nephrectomy Volume/year			<0.0001
<25	737(82.6) /4159	824(62.2) /4065	
25-49	88(9.9) /417	273(20.6) /886	
50-99	43(4.8) /189	140(10.6) /418	
100+	24(2.7) /73	88(6.6) /190	
Hospital Location			0.4592
Rural	1128(4.2) /5236	1205(3.6) /5467	
Urban	25534(95.8) /87089	32334(96.4) /89992	
Hospital Region			0.8938
Northeast	6041(22.6) /17794	8283(24.5) /19444	
Midwest	7309(27.3) /24490	9413(27.9) /26002	
South	7699(28.8) /29451	9983(29.6) /31888	
West	5692(21.3) /20876	6104(18.1) /18747	
Teaching			0.4759
Non-teaching	6482(24.3) /31172	8920(26.6) /31383	
Teaching	20180(75.7) /61153	24619(73.4) /64075	
Median Length of Stay (IQR), days	3.1(2.1-4.4)	2.7(1.7-3.9)	<0.0001
Median Total Charges for Stay (IQR), $	35223(24931–49394)	38381(27440–55923)	<0.0001
Died during hospitalization	45(0.2) /703	50(0.1) /774	0.8318

Regarding ethnicity/racial demographics, increased PN were noted in Caucasian, African-American, Hispanic, and Asian patients from 28.9%, 28.4%, 25.1%, 26.1% before the guidelines and 35.6%, 33.3%, 32.7%, and 32.3% after Guideline publication, respectively (p < 0.001). The largest improvement was 7.6% occurring in the Hispanic population. Proportionally, more PN were performed in patients with diabetes (20.8% vs. 23.3%; p = 0.005) and obesity (12.8% vs. 15.1%; p = 0.011). Less PN were performed at hospitals that performed less than 25 renal surgeries per year (82.6% vs. 62.2%; p < 0.0001). Despite the improvement length of stay in the hospital (3.1 days vs. 2.7 days; p < 0.001), the expense of PN increased by $3,158 (USD; p < 0.0001).

### Population characteristics for partial nephrectomy vs. radical nephrectomy

We identified many discrepancies between the demographics of patients who received a RN compared to a PN (Table 2). Non-modifiable factors for radical nephrectomy patients would include age and multiple comorbidities (both p < 0.001). We show social factors may play role in surgical preference because hospital location, hospital volume, and patient wealth (insurance and zip code) were all highly significant factors in our study.

**Table 2:** **Patient demographics comparing partial nephrectomy and radical nephrectomy over the 4 year study period**

Demographics	Partial Nephrectomy	Radical Nephrectomy	***p-value***
	***N = 61693***	***N = 130078***	
Mean Age ± SE	58.3 ± 0.2	60.3 ± 0.2	<0.0001
Age Group			<0.0001
<60	31251(50.7)	59090(45.4)	
≥60	30441(49.3)	70988(54.6)	
Gender			0.8440
Male	35572(57.9)	74922(57.7)	
Female	25917(42.1)	54832(42.3	
Race			0.6064
Caucasian	40245(65.2)	83623(64.3)	
African-American	5249(8.5)	11570(8.9)	
Hispanic	4029(6.5)	9705(7.5)	
Asian	1007(1.6)	2425(1.9)	
Other	11162(18.1)	22755(17.5)	
Income (Zip Code)			<0.0001
<$50,000	27052(44.9)	64633(50.6)	
≥$50,000	33242(55.1)	62976(49.4)	
Hypertension	35436(57.4)	80297(61.7)	<0.0001
Diabetes	13701(22.2)	29806(22.9)	0.1767
Obesity	8673(14.1)	15687(12.1)	<0.0001
Chronic Kidney Disease	4683(7.6)	21060(16.2)	<0.0001
CKD Stages			<0.0001
Stage 1	40(0.9)	60(0.3)	0.6139
Stage 2	206(4.4)	586(2.8)	0.0421
Stage 3	1246(26.6)	3144(14.9)	0.0004
Stage 4	350(7.5)	1075(5.1)	0.0009
Stage 5 / ESRD	40(0.8)	306(1.5)	<0.0001
Stage unspecified	2800(59.8)	15890(75.4)	<0.0001
Charlson Comorbidity Index			<0.0001
0	37308(60.5)	67959(52.2)	
1	15728(25.5)	29566(22.7)	
2	4323(7.0)	10636(8.2)	
3+	4334(7.0)	21916(16.8)	
Insurance			<0.0001
Private	34022(55.3)	57101(44.0)	
Medicare	20435(33.2)	55609(42.8)	
Medicaid	3575(5.8)	8684(6.7)	
Other	3531(5.7)	8387(6.5)	
Hospital Bed Number			0.1792
Small	5428(9.0)	11678(9.2)	
Medium	11508(19.1)	28038(22.0)	
Large	43265(71.9)	87867(68.8)	
Hospital Nx Volume/year			<0.0001
<25	1560(70.4)	6663(81.5)	
25-49	361(16.3)	942(11.5)	
50-99	184(8.3)	424(5.2)	
100+	112(5.1)	150(1.8)	
Hospital Location			<0.0001
Rural	2333(3.9)	8371(6.6)	
Urban	57868(96.1)	119212(93.4)	
Hospital Region			0.0009
Northeast	14324(23.7)	22913(17.9)	
Midwest	16723(27.6)	33770(26.3)	
South	17683(29.2)	43656(34.1)	
West	11795(19.5)	27828(21.7)	
Teaching			<0.0001
Non-teaching	15401(25.6)	47154(37.0)	
Teaching	44800(74.4)	82429(63.0)	
Median Length of Stay (IQR), days	2.9(1.9-4.2)	3.4(2.2-5.2)	<0.0001
Median Total Charges for Stay (IQR), $	36832(26298–53058)	37359(25669–59124)	<0.001
Died during hospitalization	93(0.2)	1384(1.1)	<0.0001

### Multivariate analysis of pre and post AUA T1 renal mass guidelines publication

Using logistic regression analysis, patients had a 24% increased odds of having a PN after Guideline publication (OR 1.242 95%CI 1.001-1.542; p = 0.0489) More PN were performed on obese patients (OR 1.527 95%CI 1.113-2.058; p = 0.0054). Lastly, patients who had surgery at a hospital that does at least 25 nephrectomies (partial or radical) had a 2-fold higher odds of having a PN after Guideline publication than prior (p < 0.0001)

### Trends of partial nephrectomies in relation to guideline release

**Figure 1:**
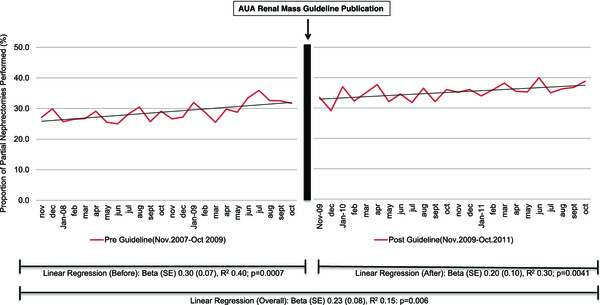
**Incidence of PN performed as a proportion of nephrectomies (radical and partial) performed in the United States Nationwide Inpatient Sample from November 2007 to October 2011 noting when the T1a Renal AUA Guidelines Recommendation publication date.**

The overall proportion of PN performed from increased significantly throughout the study period (R^2^ = 0.24; p = 0.0005; Figure 1). We divided this trend into two separate linear regression lines to determine the rate of change of PN performed prior to Guideline publication (R^2^ = 0.40 p = 0.0007) and after Guideline publication (R^2^ = 0.30 p = 0.0041). We then compared the interaction between the regression lines noting they were not statistically different (p = 0.4613). Figure 1 displays the trends over time. There was no increase in PN for patient with CKD over this time period (R^2^ 0.0007, p = 0.8592) or between the two time periods (R2 0.02 vs. 0.04; p = 0.2425). Linear regression could not be performed on specifically CKD stage III/IV due to low sample size leading to potentially large error.

## Discussion

**Figure 2:**
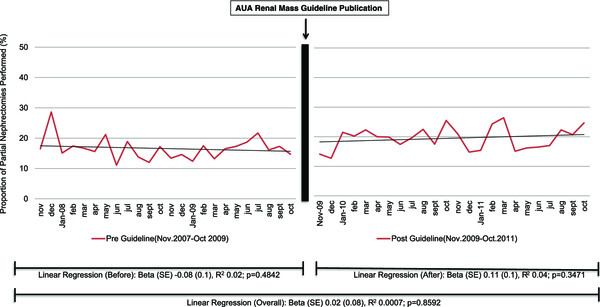
**Incidence of PN performed in patients with preoperative chronic kidney disease as a proportion of nephrectomies (radical and partial) performed in the United States Nationwide Inpatient Sample from November 2007 to October 2011 noting when the AUA Guidelines Recommendation Release.**

We noted a 6.4% absolute increase in the proportion of PN in a two-year period performed before and after the guideline release; however, PN remains only about 35% of all nephrectomies performed. We used an adjusted multivariable analysis to identify a 23% increased odds of having a PN in the 2 years after Guidelines publication (Table 3). We were expecting to identify a rise in the rate of PN after Guideline publication, however there was no difference in incidence (Figure 1). While increase in PN utilization increase after AUA T1 Renal Mass guidelines publication was recently reported by Bjurlin et al. who noted a one year increase in PN proportion from 27% to 32% [18], our data are longer term and the conclusions we draw while seemingly similar (i.e., an overall increase). Nonetheless our results demonstrate that increase in PN utilization was more likely due to the overall steady rise in incidence over time rather than the AUA T1 Renal Mass Guidelines making a significant immediate impact (Figure 1). We describe increases in PN for high risk patients such as those with obesity, diabetes, and hypertension. Unfortunately, we noted no change in PN proportion with preoperative non-dialysis dependent CKD before and after the AUA T1 Renal Mass Guideline publication (Figure 2). While we are unable to target specific reasoning behind the lack of increase in partial nephrectomy among non-dialysis depended CKD patients, such as tumor factors, this finding is concerning considering it is highlighted as an imperative indication of PN in the Guidelines [15]. Moreover, the AUA guideline is similar to the EAU guidelines regarding treatment of renal mass with preference toward nephron sparing surgery (initially published in June, 2007 with update in 2010) [19, 20]. We highlight this finding as a potential quality of care issue which deserves further follow up.

**Table 3:** **Multivariable analysis comparing partial nephrectomy vs. radial nephrectomy using the 2 year study period prior to publications of the T1a renal AUA guidelines recommendations as reference**

Before AUA T1 Renal Guideline (reference) vs. After AUA T1 Renal Guideline				
	Odds Ratio	95% Wald Confidence Limits	P	Overall P
Partial Nephrectomy vs. Radical Nephrectomy (reference)	1.242	1.001 - 1.542	0.0489	<0.0001
Obesity Yes vs. No (reference)	1.527	1.133 - 2.058	0.0054	
Nephrectomy Hospital Volume Less than 25 cases (reference)			<0.0001	
Volume 25–49 cases	2.09	1.600 -2.731		
Volume 50–99 cases	2.222	1.535 - 3.217		
Volume 100+ cases	2.566	1.430 - 4.605

Patients presenting with a renal mass and concurrent CKD are at highest risk of renal related morbidity and mortality [21–23]. However, our study shows the United States continues to struggle to provide PN to patients because of social issues such as the patient’s financial means and where they reside, which agrees with reports from other reports from the NIS [24] and SEER Medicare data [24]. Recent health reform in American healthcare may change these trends and should be followed over time [25]. Despite current changes in healthcare, issues such as access to care, appropriate training, and hospital resources are also concerns for urologists. For example, urologists were criticized for poor adherence to guideline recommendations regarding a lower than anticipated use of intravesical chemotherapy for non-muscle invasive bladder cancer in population studies [26]. However when thoroughly investigated to improve care, the investigators found that the initial the baseline rates of non-adherence where actually justified [27]. The true reasons for not performing a PN also need to be studied in a similar fashion to the studies regarding non-muscle invasive bladder cancer, potentially a utilization of the newly developed American Urologic Association Quality Registry (AQUA).

Another potential reason for lack of increased adoption of PN for T1 renal neoplasms is that the seemingly overwhelming and robust retrospective data supporting PN has been called into question by publication of only prospective randomized randomized trial to address this topic, EORTC 30904, which was published in April 2011. This trial randomized 541 patients [PN (n = 268)/RN (n = 273)], with a median follow-up of 9.3 years, and noted that overall survival with a hazard ratio (HR) of 1.50, testing for non-inferiority was not significant (p = 0.77), and test for superiority was significant (p = 0.03) in favor for RN, though in RCC patients there were no significant differences with respect to overall survival. These findings were presented cautiously given shortcomings in study design and execution [13], however, they have already generated a great deal of controversy amongst thought leaders [18, 22, 23]. For the observation period and our analysis however, the impact of this study and its findings are likely limited and not noted. Indeed, longer observation is requisite to evaluate the impact of this study both on practice patterns in the United States, as well as in the formulation of updates to the AUA Guidelines document.

The criticism for PN utilization of only 35% in this population based cohort is tempered by the overall rise in incidence of PN in the past 4 years as well as the lack of evidence investigating physician clinical decision-making. We identified the largest improvement in PN performed based on hospital volume. In multivariable analysis, patients had a 2 fold increased odds chance of having a PN if the hospital performs at least 25 renal cases (partial or radical nephrectomies) per year confirming results of prior studies using the NIS [28]. Various reasons may account for this increase. Some reasons for the change in volume may include lower volume hospitals increasing number of PN performed, reflection of current referral patterns, and a combination of both. Additionally, PN in obese patients increased as which most likely is reflecting the increasing obesity epidemic in the United States, which is likely a welcome development given the fact that obesity is a driver for CKD as well [29].

We performed this study not to determine causality of the AUA T1 Renal Mass Guidelines on PN in the United States, but to identify potential opportunities for improvement. Creation of a guideline should only be the beginning of a continuous process of improvement as the ultimate success of depends on critical evaluation over time [30]. In general, guidelines are data driven documents that provide assistance to physicians and come after a majority of the physicians have already adopted the practice [31]. PN has been performed for more than a century, yet continued debate remains regarding its benefit and utilization [13, 32, 33]. The adoption of PN may be due in part to changes in technology such as laparoscopy and robotic surgery [4, 7]. Indeed our findings that the median hospital length of stay has decreased while there may be a rise in overall cost of procedure for PN, while likely indicative increasing adoption of minimally invasive partial nephrectomy (laparoscopic or robotic) our group has reported in previous studies [34, 35].

Limitations in the data derived from national databases are only as accurate as the codes that have been chosen for the patient. The NIS database does not have pathologic data and therefore may not solely focus on cT1a renal cortical masses. However, the NIS was chosen for its strength in the documentation of the procedure itself as well as the fact that up to 25% of small renal masses have benign histology, and would therefore not be documented in oncology driven databases [15]. Moreover, the pathology of the renal mass is usually not available to the surgeon prior to the decision for type of surgery, and we sought to examine trends in procedure utilization as opposed to oncologic outcomes. Though only 4 years are evaluated in our analysis, it is possible that tumor stage may have changed over time and could confound the analysis. We have provided extensive exclusion criteria to remove patients with advanced tumors from our analysis, which have been validated by our group and others in previous analyses [6, 20, 24]. Furthermore, CKD can be a generalized term and we did note variation within the CKD stages 2–5 codes. Nonetheless, the rate of CKD in patients undergoing RN or PN was 4.4% overall, which is similar in rate to the 4.3% prevalence of CKD Stage 1–4 reported in medicare patients and suggests that there is no coding bias favoring reporting of dialysis dependent CKD over CKD Stages 1–4. More specific and accurate determination and documentation of CKD are needed for patients presenting for PN for appropriate counseling.

Partial nephrectomy utilization has increased over the 4 years selected for evaluation and should be recognized. However, the lingering question is how many present with amenable tumors and/or various indications for partial nephrectomy and do not have the option offered. One such patient’s are those with CKD, in which a surgical decision could have significant impact on future health in which the urologists is not likely to serve the penalty. Therefore, a concerted effort should be placed on identifying patients who would most benefit from partial nephrectomy and providing that service despite demographic challenges. Additionally, further investigation regarding current clinical decision may provide more immediate problem solving opportunities.

## Conclusions

We have identified a steady increase in PN over the four-year study period and more PN performed after the publication of the AUA Clinical T1 Renal Mass Treatment Guidelines. However, the rate of PN performed after the guidelines release was not faster indicating that other factors may be influencing the adoption of PN. Additional studies and longer-term follow up are needed to determine the practical effects and clinical significance of evidence-based guidelines.

## References

[1] Robson CJ, Churchill BM, Anderson W (1969). The results of radical nephrectomy for renal cell carcinoma. J Urol.

[2] Chow WH, Devesa SS, Warren JL, Fraumeni JF (1999). Rising incidence of renal cell cancer in the United States. JAMA.

[3] Kane CJ, Mallin K, Ritchey J, Cooperberg MR, Carroll PR (2008). Renal cell cancer stage migration: analysis of the national cancer data base. Cancer.

[4] Hollenbeck BK, Taub DA, Miller DC, Dunn RL, Wei JT (2006). National utilization trends of partial nephrectomy for renal cell carcinoma: a case of underutilization?. Urology.

[5] Colli J, Sartor O, Grossman L, Lee BR (2012). Underutilization of partial nephrectomy for stage t1 renal cell carcinoma in the United States, trends from 2000 to 2008. A long way to go. Clin Genitourin Cancer.

[6] Cooperberg MR, Mallin K, Kane CJ, Carroll PR (2011). Treatment trends for stage I renal cell carcinoma. J Urol.

[7] Tanagho YS, Figenshau RS, Sandhu GS, Bhayani SB (2012). Is there a financial disincentive to perform partial nephrectomy?. J Urol.

[8] Bagrodia A, Darwish OM, Rapoport Y, Margulis V (2012). Risk prediction in the management of small renal masses. Curr Opin Urol.

[9] Malcolm JB, Bagrodia A, Derweesh IH, Mehrazin R, Diblasio CJ, Wake RW, Wan JY, Patterson AL (2009). Comparison of rates and risk factors for developing chronic renal insufficiency, proteinuria and metabolic acidosis after radical or partial nephrectomy. BJU Int.

[10] Weight CJ, Larson BT, Gao T, Campbell SC, Lane BR, Kaouk JH, Gill IS, Klein EA, Fergany AF (2010). Elective partial nephrectomy in patients with clinical T1b renal tumors is associated with improved overall survival. Urology.

[11] Weight CJ, Lieser G, Larson BT, Gao T, Lane BR, Campbell SC, Gill IS, Novick AC, Fergany AF (2010). Partial nephrectomy is associated with improved overall survival compared to radical nephrectomy in patients with unanticipated benign renal tumours. Eur Urol.

[12] Woldrich J, Mehrazin R, Bazzi WM, Bagrodia A, Kopp RP, Malcolm JB, Kane CJ, Patterson AL, Wan JY, Derweesh IH (2012). Comparison of rates and risk factors for development of anaemia and erythropoiesis-stimulating agent utilization after radical or partial nephrectomy. BJU Int.

[13] Van Poppel H, Da Pozzo L, Albrecht W, Matveev V, Bono A, Borkowski A, Marechal JM, Klotz L, Skinner E, Keane T, Claessens I, Sylvester R (2007). A prospective randomized EORTC intergroup phase 3 study comparing the complications of elective nephron-sparing surgery and radical nephrectomy for low-stage renal cell carcinoma. Eur Urol.

[14] MacLennan S, Imamura M, Lapitan MC, Omar MI, Lam TB, Hilvano-Cabungcal AM, Royle P, Stewart F, MacLennan G, MacLennan SJ, Canfield SE, McClinton S, Leyshon Griffiths TR, Ljungberg B, N’Dow J (2012). Systematic review of oncological outcomes following surgical management of localised renal cancer. Eur Urol.

[15] Campbell SC, Novick AC, Belldegrun A, Blute ML, Chow GK, Derweesh IH, Faraday MM, Kaouk JH, Leveillee RJ, Matin SF, Russo P, Uzzo RG (2009). Guideline for management of the clinical T1 renal mass. J Urol.

[16] Kates M, Whalen MJ, Badalato GM, McKiernan JM (2013). The effect of race and gender on the surgical management of the small renal mass. Urol Oncol.

[17] Patel HD, Kates M, Pierorazio PM, Allaf ME (2014). Race and sex disparities in the treatment of older patients with T1a renal cell carcinoma: a comorbidity-controlled competing-risks model. Urol Oncol.

[18] Bjurlin MA, Walter D, Taksler GB, Huang WC, Wysock JS, Sivarajan G, Loeb S, Taneja SS, Makarov DV (2013). National trends in the utilization of partial nephrectomy before and after the establishment of AUA guidelines for the management of renal masses. Urology.

[19] Ljungberg B, Cowan NC, Hanbury DC, Hora M, Kuczyk MA, Merseburger AS, Patard JJ, Mulders PF, Sinescu IC, European Association of Urology Guideline G (2010). EAU guidelines on renal cell carcinoma: the 2010 update. Eur Urol.

[20] Ljungberg B, Hanbury DC, Kuczyk MA, Merseburger AS, Mulders PF, Patard JJ, Sinescu IC, European Association of Urology Guideline Group for renal cell c (2007). Renal cell carcinoma guideline. Eur Urol.

[21] Go AS, Chertow GM, Fan D, McCulloch CE, Hsu CY (2004). Chronic kidney disease and the risks of death, cardiovascular events, and hospitalization. N Engl J Med.

[22] Demirjian S, Lane BR, Derweesh IH, Takagi T, Fergany A, Campbell SC (2014). Chronic kidney disease due to surgical removal of nephrons: relative rates of progression and survival. J Urol.

[23] Smaldone MC, Egleston B, Uzzo RG, Kutikov A (2012). Does partial nephrectomy result in a durable overall survival benefit in the Medicare population?. J Urol.

[24] Becker A, Roghmann F, Trinh QD, Hansen J, Tian Z, Shariat SF, Noldus J, Perrotte P, Graefen M, Karakiewicz PI, Sun M (2013). Sociodemographic disparities in the treatment of small renal masses. BJU Int.

[25] Rosenbaum L, Shrank WH (2013). Taking our medicine–improving adherence in the accountability era. N Engl J Med.

[26] Chamie K, Saigal CS, Lai J, Hanley JM, Setodji CM, Konety BR, Litwin MS, Urologic Diseases in America P (2011). Compliance with guidelines for patients with bladder cancer: variation in the delivery of care. Cancer.

[27] Barocas DA, Liu A, Burks FN, Suh RS, Schuster TG, Bradford T, Moylan DA, Knapp PM, Murtagh DS, Morris D, Dunn RL, Montie JE, Miller DC (2013). Practice based collaboration to improve the use of immediate intravesical therapy after resection of nonmuscle invasive bladder cancer. J Urol.

[28] Sun M, Bianchi M, Trinh QD, Abdollah F, Schmitges J, Jeldres C, Shariat SF, Graefen M, Montorsi F, Perrotte P, Karakiewicz PI (2012). Hospital volume is a determinant of postoperative complications, blood transfusion and length of stay after radical or partial nephrectomy. J Urol.

[29] Hedley AA, Ogden CL, Johnson CL, Carroll MD, Curtin LR, Flegal KM (2004). Prevalence of overweight and obesity among US children, adolescents, and adults, 1999–2002. JAMA.

[30] Basinski AS (1995). Evaluation of clinical practice guidelines. CMAJ.

[31] Dahm P, Yeung LL, Gallucci M, Simone G, Schunemann HJ (2009). How to use a clinical practice guideline. J Urol.

[32] Novick AC, Derweesh I (2005). Open partial nephrectomy for renal tumours: current status. BJU Int.

[33] Tan HJ, Norton EC, Ye Z, Hafez KS, Gore JL, Miller DC (2012). Long-term survival following partial vs radical nephrectomy among older patients with early-stage kidney cancer. JAMA.

[34] Parsons JK, Palazzi K, Chang D, Stroup SP (2013). Patient safety and the diffusion of surgical innovations: a national analysis of laparoscopic partial nephrectomy. Surg Endosc.

[35] Woldrich JM, Palazzi K, Stroup SP, Sur RL, Parson JK, Chang D, Derweesh IH (2013). Trends in the surgical management of localized renal masses: thermal ablation, partial and radical nephrectomy in the USA, 1998–2008. BJU Int.

[CR36] The pre-publication history for this paper can be accessed here:http://www.biomedcentral.com/1471-2490/14/101/prepub

